# Glucosamine protects against neuronal but not vascular damage in experimental diabetic retinopathy

**DOI:** 10.1016/j.molmet.2021.101333

**Published:** 2021-09-20

**Authors:** Rachana Eshwaran, Matthias Kolibabka, Gernot Poschet, Gregor Jainta, Di Zhao, Loic Teuma, Katharina Murillo, Hans-Peter Hammes, Martina Schmidt, Thomas Wieland, Yuxi Feng

**Affiliations:** 1 Experimental Pharmacology Mannheim, European Center for Angioscience, Medical Faculty Mannheim, Heidelberg University, Mannheim, Germany; 2 5th Medical Clinic, Medical Faculty Mannheim, Heidelberg University, Mannheim, Germany; 3 Center for Organismal Studies (COS), Heidelberg, Germany; 4 University of Groningen, Department of Molecular Pharmacology, 9713AV, Groningen, the Netherlands; 5 University of Groningen, University Medical Center Groningen, Groningen Research Institute for Asthma and COPD (GRIAC), Groningen, the Netherlands; 6 DZHK (German Center for Cardiovascular Research), Partner site Heidelberg/Mannheim, Germany

**Keywords:** Retina, Neuronal, Vascular damage, Endothelial cells, Müller cells

## Abstract

**Objective:**

Glucosamine, an intermetabolite of the hexosamine biosynthesis pathway (HBP), is a widely used nutritional supplement in osteoarthritis patients, a subset of whom also suffer from diabetes. HBP is activated in diabetic retinopathy (DR). The aim of this study is to investigate the yet unclear effects of glucosamine on DR.

**Methods:**

In this study, we tested the effect of glucosamine on vascular and neuronal pathology in a mouse model of streptozotocin-induced DR *in vivo* and on cultured endothelial and Müller cells to elucidate the underlying mechanisms of action *in vitro*.

**Results:**

Glucosamine did not alter the blood glucose or HbA_1c_ levels in the animals, but induced body weight gain in the non-diabetic animals. Interestingly, the impaired neuronal function in diabetic animals could be prevented by glucosamine treatment. Correspondingly, the activation of Müller cells was prevented in the retina as well as in cell culture. Conversely, glucosamine administration in the normal retina damaged the retinal vasculature by increasing pericyte loss and acellular capillary formation, likely by interfering with endothelial survival signals as seen *in vitro* in cultured endothelial cells. Nevertheless, under diabetic conditions, no further increase in the detrimental effects were observed.

**Conclusions:**

In conclusion, the effects of glucosamine supplementation in the retina appear to be a double-edged sword: neuronal protection in the diabetic retina and vascular damage in the normal retina. Thus, glucosamine supplementation in osteoarthritis patients with or without diabetes should be taken with care.

## Highlights

• The hexosamine biosynthesis pathway (HBP) is activated in diabetic retinopathy (DR), which manifests as vascular and neuronal damage in the retina.
• Glucosamine, metabolized in the HBP, is a widely used oral supplement for osteoarthritis treatment.
• Glucosamine supplementation improved neuronal function in retinas of mice with experimental DR, but induced vascular damage in normal retinas.
• Müller cell activation and endothelial survival signals in the retina were affected by glucosamine.
• Patients with or without diabetes should take caution with glucosamine supplementation.

## Abbreviations

AC: Acellular capillaries
Ang2: Angiopoietin 2
DR: Diabetic retinopathy
EC: Endothelial cell
ERG: Electroretinogram
GFAP: Glial fibrillary acidic protein
GOT: Glutamic oxaloacetase transaminase
GPT: Glutamic pyruvic transaminase
HBP: Hexosamine biosynthesis pathway
HRMVECs: Human retinal microvascular endothelial cells
HUVECs: Human umbilical vein endothelial cells
O-GlcNAc: O-linked N-Acetylglucosamine
OCT: Optical coherence tomography
PC: Pericyte
rMCs: Rat Müller cell line;
UDP-GlcNAc: Uridine diphosphate N-Acetyl glucosamine;
VEGF: Vascular endothelial growth factor
VEGFR2: Vascular endothelial growth factor receptor 2

## 1 Introduction

The hexosamine biosynthesis pathway (HBP), a nutrient-sensing pathway, accounts for 2–5% of the total glucose flux under normal conditions [1] and combines elements of glucose, amino acid, fatty acid, and nucleotide metabolisms. The final product of the pathway is UDP-GlcNAc, which is the substrate for protein O-GlcNAcylation, a post-translational protein modification occurring at serine and threonine residues. Activation of the HBP and protein O-GlcNAcylation are considered key mediators in the initiation and progression of diabetic complications such as diabetic retinopathy and diabetic nephropathy [2,3].

Diabetic retinopathy is a long-term microvascular complication of diabetes. In its early stage, diabetic retinopathy is characterized morphologically by the loss of pericytes and subsequent disappearance of endothelial cells from capillaries. This leads to the formation of acellular capillaries that consist of only the vascular basement membrane and do not support blood flow, resulting in the progression of the disease [4]. The pathology of diabetic retinopathy is exemplified not only by vascular damage, but also neuronal damage. Some studies have shown impaired neuroretinal function prior to vascular lesions in humans [[5], [6], [7]], observed along with the activation of glial cells, which is indicated by increased expression of Glial Fibrillary Acidic Protein (GFAP). The hyperglycemia-induced vascular and neuronal damage in the retina is modulated by the interplay of oxidative stress, inflammation, and aberrant growth factor signaling via VEGF-A and Angiopoietin-2 (Ang2) [[8], [9], [10]].

Biochemically, glucosamine is an intermetabolite of the HBP, an offshoot of glycolysis in glucose metabolism. It stimulates glycosaminoglycan production and the incorporation of sulfur into cartilage [11]. For this reason, glucosamine has been extensively studied in the context of osteoarthritis and joint pain. Several clinical studies conducted on oral supplementation of glucosamine sulfate demonstrate its efficacy in slowing down the progression of osteoarthritis [12] and increasing the rate of cartilage renewal [13]. Glucosamine is currently the oral supplement most widely used by osteoarthritis patients. It can act as a substrate in the production of cartilage components and inhibit Il-1β–correlated inflammatory response [14]. Additionally, glucosamine is an effective scavenger of superoxide radicals, inhibiting oxidative stress–induced apoptosis in retinal ganglion cells [15,16]. Furthermore, glucosamine exhibits antiangiogenic properties by hampering the signaling mediated by growth factors. Thus, glucosamine exerts several beneficial effects on oxidative stress, inflammation, and growth factor signaling, hypothetically countering the pathological mechanisms in diabetic retina. The implication of exogenous glucosamine in glucose metabolism, however, is controversial. Exogenous glucosamine application increases protein O-GlcNAcylation, but whether this promotes progression of the disease or is still protective despite the increase in protein O-GlcNAcylation is a matter of debate [17,18]. Due to the ubiquitous presence of the HBP and protein O-GlcNAcylation in mammalian cells and the additional regulation via other signaling pathways [19,20], exogenous glucosamine supply could cause different effects in different cell types.

Diabetes and osteoarthritis often occur concomitantly in elderly patients. Additionally, exogenous glucosamine supply, though it ameliorates cartilage damage, might increase protein O-GlcNAcylation in the retina and thus aggravate diabetic retinopathy. Therefore, we sought to determine the effects of oral glucosamine supplementation in an experimental diabetic retinopathy model in mice and further elucidate the mechanisms of action by studying its effects on endothelial and Müller cells *in vitro*.

## 2 Materials and methods

### 2.1 Animals

The use of mice in this study was approved by the local ethics committee (G178/15, Regierungspräsidium Karlsruhe, Germany). The care and experimental use of the animals were in accordance with institutional guidelines, in compliance with the Association for Research in Vision and Ophthalmology (ARVO) statement, and reported in compliance with the ARRIVE guidelines. The mice were housed in a 12-hour dark/light cycle with unrestricted access to food and water and under the supervision of trained staff. Diabetes induction in the mice was performed via intraperitoneal injection of streptozotocin (145 mg/kg body weight) into 8-week-old male C57Bl/6 mice, and glucosamine treatment was commenced after 1 week for up to 24 weeks. Glucosamine was provided to the animals via incorporation into food at a concentration of 10 g/kg [21]. Metabolic parameters of the mice were assessed using a metabolic cage for 16 h. At the end of the experiment, the mice were euthanized by ketamine and xylazine administered intraperitoneally, followed by cervical dislocation. The eyes were collected and stored at −80 °C for further analysis. Glucosamine concentration in the plasma was determined via UPLC separation as described below, and hepatic enzymes were investigated to establish the safety of glucosamine administration.

### 2.2 Analysis of glucosamine plasma concentration

Non–thiol-containing amino acids were quantified after specific labeling with the fluorescence dye AccQ-TagTM (Waters) according to the manufacturer's protocol. The resulting derivatives were separated by reversed phase chromatography on an Acquity BEH C18 column (150 mm × 2.1 mm, 1.7 μm, Waters) connected to an Acquity H-class UPLC system and quantified by fluorescence detection (Acquity FLR detector, Waters). The column was heated to 42 °C and equilibrated with 5 column volumes of buffer A (140 mM sodium acetate pH 6.3, 7 mM triethanolamine) at the flow rate of 0.45 mL min^-1^. Baseline separation of amino acid derivates was achieved by increasing the concentration of acetonitrile (B) in buffer A as follows: 1 min 8% B, 7 min 9% B, 7.3 min 15% B, 12.2 min 18% B, 13.1 min 41% B, 15.1 min 80% B, hold for 2.2 min, and return to 8% B for 1.7 min. Data acquisition and processing were performed with the Empower3 software suite (Waters).

### 2.3 Retina digestion and quantitative morphometry

Retinal digestion was performed to isolate retinal vessels, and PAS staining was done to quantify the retinal cells as previously described [22] using the following procedure: The eyes were fixed in 4% formalin for 48 h, after which the retinas were isolated under the microscope. The isolated retinas were incubated in water at 37 °C for 30 min and subsequently incubated at 37 °C for 3 h in 3% trypsin dissolved in Tris–HCl buffer (pH 7.0). The retinas were transferred onto a glass object slide and carefully washed with drops of water until the retinal vasculature was visible under the microscope. The vasculature was then dried on the glass object slide and then stained with Period-Acid-Schiff (PAS) and Mayer's Hamalaun staining. The slides were further embedded with Entellan® and observed under the microscope, and photos of 40 × magnification were taken. The numbers of pericytes and acellular capillaries were quantified. For quantification of pericytes, 10 microscopic fields of 40 × magnification were randomly selected, the pericytes as well as endothelial cells in the vasculature were identified, and the software AnalysisPro (Olympus) was used to record the cell numbers and calculate the capillary area. The acellular capillaries were quantified using an integration ocular with a grid of 100 squares. The numbers of squares containing acellular capillary segments were counted in 10 randomly selected microscopic fields.

### 2.4 Cell culture

The use of Human Umbilical Vein Endothelial Cells (HUVECs) was approved by the local ethics committee (Medical Faculty Mannheim, Heidelberg University, Germany). HUVECs were isolated from umbilical cords obtained from healthy newborns with the informed consent of their mothers using a previously described protocol [23]. HUVECs were cultured in Endothelial Cell Basal Medium (ECBM, Promocell) supplemented with 10% fetal calf serum (FCS) and 1% Penicillin-Streptomycin (PS) on 1% gelatin-coated flasks. HUVECs were used in experiments between passage 1 and 3. Human Retinal Microvascular Endothelial Cells (HRMVECs) (PB-CH-160-8511, Pelobiotech, Germany) were cultured on 1% gelatin-coated flasks in ECBM MV, specific for microvascular cells, supplemented with 10% FCS and 1% PS. Rat Müller cell line (rMCs) was obtained from the lab of Prof. Ingrid Fleming, Goethe Institute, Frankfurt, and cultured in DMEM supplemented with 200 mM glutamine, 10% FCS, and 1% PS. The cells were stimulated with high glucose at a concentration of 30 mM by dissolving the appropriate weight of d-Glucose in ECBM/ECBM MV or DMEM for HUVECs/HRMVECs and Müller cells, respectively. Glucosamine stimulation in the cells was performed by preparing a stock solution of 100 mM glucosamine (d-Glucosamine Hydrochloride, G4875 Sigma–Aldrich) in ECBM/DMEM immediately prior to stimulation and diluting according to the required concentration in the experiments.

### 2.5 Multifocal electroretinogram (mfERG) and optical coherence tomography (OCT)

Multifocal electroretinography (mfERG) was performed as previously described [24]. The mice were placed in front of the scanning laser ophthalmoscope (sLO) device (RETImap, Roland Consult, Brandenburg an der Havel, Germany), with a DTL electrode placed at the cornea. Subcutaneous silver needle electrodes were positioned at the neck of the mice, serving as reference and ground electrodes. A 90-dioptrie contact lens mounted over viscous 2% methocel gel was placed on the eyes of the mice. An array of 7 equally sized hexagons was chosen and stimulation was performed using 150 cd/m^2^ and 1 cd/m^2^ for the m-sequence with four dark frames in between the stimuli. An average of eight cycles was used for each hexagon for the final analyses, which were processed by the built-in 50-Hz band filter to reduce background noise. For each animal, the average amplitude of the 6 hexagons around the optic nerve head was used for final analyses. mfERG recording took place under photopic conditions wherein both rod and cone photoreceptors were activated in mice. The initial negative-going N1-wave was initiated by photoreceptors, whereas the following positive-going P1-wave was generated in the inner retina, mainly by ON-bipolar cells under the influence of Müller cells. Optical coherence tomography (OCT) was subsequently performed using the built-in OCT of the RETImap system. Retinal thickness was measured at five locations along the border between the inner third and the outer two-thirds of the retina.

### 2.6 Whole retina immunofluorescence staining

The eyes were fixed in 4% paraformaldehyde on ice for 2 h. The retinas were dissected and washed in PBS for 3 h and incubated in permeabilization/blocking solution (3% w/v BSA and 0.1% Triton-X in PBS) for 1.5 h at room temperature. The retinas were incubated with primary antibody (GFAP, Dako Z0334, diluted 1:500 in PBS) overnight at 4 °C. Post-incubation, the retinas were washed with PBS for 3 h and incubated with the secondary antibody solution (swine anti-rabbit FITC F0205, Dako, diluted 1:20 in PBS) for 1.5 h at room temperature. The retinas were subsequently washed with PBS, cut into 4 leaves, mounted on glass slides using Roti FluorCare mounting medium, and covered with a coverslip. The retinas were imaged using a Leica SP8 confocal microscope. Four fields per retina were randomly chosen and z-stack images spanning 80 μm were obtained. The images were visualized using a 3D viewer, displaying the GFAP staining in astrocytes on the superficial layer and in Müller cells spanning the depth of the retina. Astrocytes were quantified visually in the superficial layer, and the lengths of the GFAP staining in individual Müller cells were quantified per field and compared between groups.

### 2.7 Immunofluorescence staining in HUVECs and rMCs

HUVECs and rMCs were cultured according to experimental conditions and subsequently washed with PBS and fixed with 4% Histofix for 10 min, after which they were incubated with a permeabilization/blocking solution (2.5% BSA and 0.3% Triton-X in PBS) for 1 h at room temperature. The cells were further incubated with 200 μL of primary antibody solution (VEGFR2 55B11, Cell Signaling; Ang2 SC74403, Santa Cruz; GFAP Z0334, Dako; diluted 1:200 in PBS) overnight at 4 °C, then washed thrice with PBS and incubated with the secondary antibodies (swine anti-rabbit FITC, F0205, Dako; diluted 1:20 in PBS) for 1 h at room temperature in the dark. The cells were subsequently washed thrice with PBS, incubated with DAPI (1 μg/mL) for 10 min, and washed twice with PBS. They then were covered with Roti FluorCare mounting medium and covered with coverslips. The images were obtained using a Leica SP8 confocal microscope, and fluorescence intensity quantification was done using ImageJ.

### 2.8 Protein isolation and immunoblotting

Proteins from HUVECs and rMCs were extracted in RIPA buffer using a previously described method [25]. The primary antibodies used were: VEGFR2 (55B11 Cell Signaling, 1:1000); Ang2 (SC74403 Santa Cruz, 1:500); Tubulin (T6557 Sigma–Aldrich, 1:2000); GlcNAc (ab2739 Abcam, 1:1000); GFAP (Z0334 Dako, 1:5000); VEGF (ab16154 Abcam, 1:2000), diluted in Tris-buffered saline containing 0.1% Tween20 (TBST). Secondary antibodies against mouse (Rabbit anti-mouse peroxidase, A-9044 Sigma–Aldrich) and rabbit (Goat anti-rabbit peroxidase, A-9169 Sigma–Aldrich) were diluted 1:20,000 in TBST. The obtained images were quantified using ImageJ.

### 2.9 Statistical analysis

The data are presented as mean ± SD. Statistical analyses were performed with GraphPad Prism 6. Paired/unpaired student's t-test or one-way ANOVA with Tukey's multiple comparison test were used. p values < 0.05 were considered statistically significant.

## 3 Results

### 3.1 Glucosamine affects neither blood glucose nor HbA1c levels, but induces a body weight gain in non-diabetic mice

**Figure 1:**
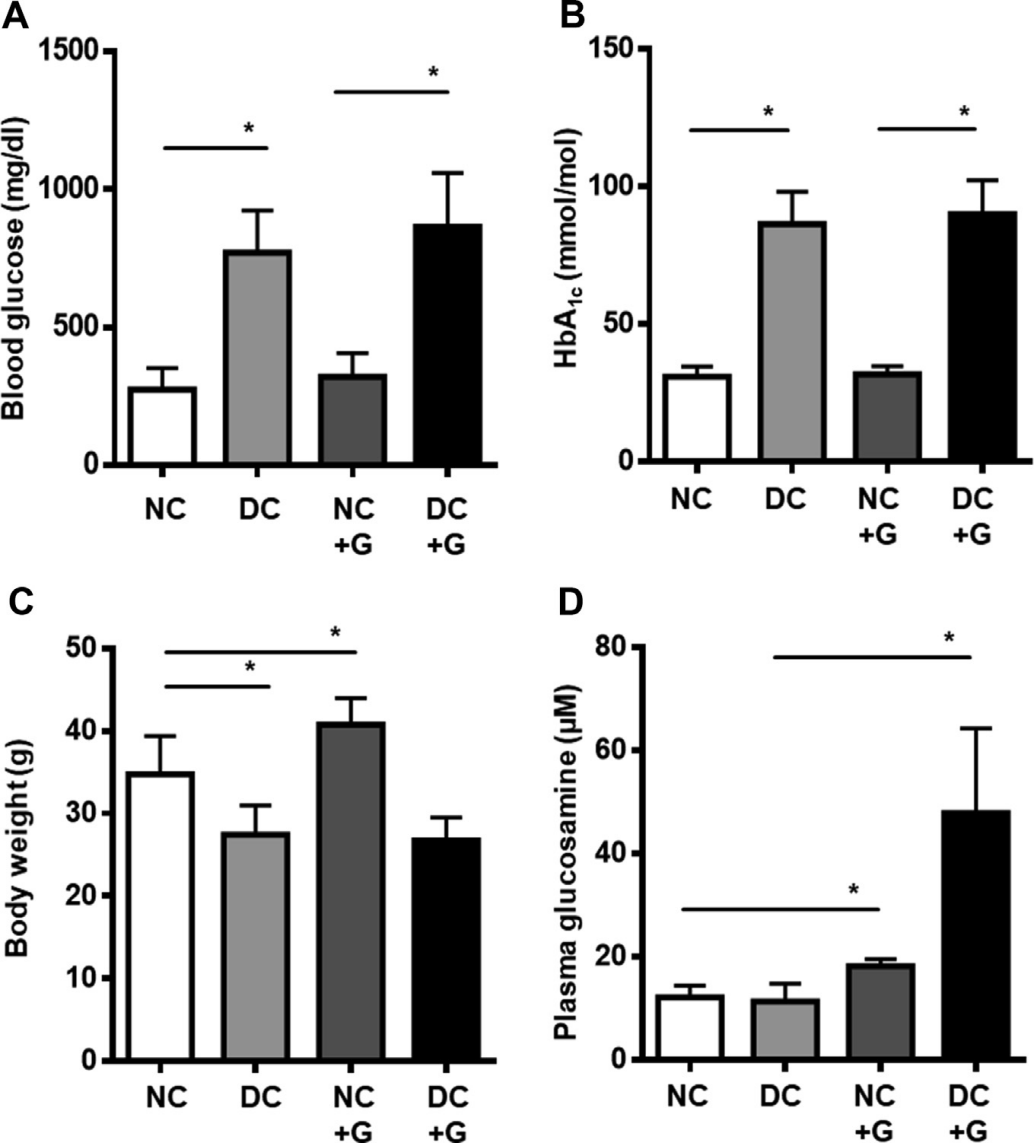
**Glucosamine does not affect blood glucose or HbA1c.** Mice without and with diabetes induction were supplemented with glucosamine (10 g/kg food) for 24 weeks and analyzed for the following parameters. **A:** Blood glucose. **B:** HbA1c. **C:** Body weight: diabetic animals show a reduction in weight, while glucosamine treatment induced a gain in non-diabetic animals. **D:** Glucosamine levels in the plasma. n = 6–8, ∗p < 0.05.

To assess the effect of glucosamine on the glucose metabolism of the mice, the level of blood glucose was first measured using a glucometer. Compared to healthy controls, a consistent increase in the levels of blood glucose in streptozotocin-injected diabetic animals was observed. Glucosamine supplementation in the food did not alter the blood glucose levels of non-diabetic and diabetic animals compared to their respective controls (Figure 1A). HbA_1c_ levels reflect the average level of blood sugar over the past 120 or 40 days in humans and mice, respectively. Correspondingly, (Figure 1B) streptozotocin-induced hyperglycemia significantly elevated HbA1c levels measured over the course of the experiment in the diabetic mice. There was no influence from glucosamine supplementation, suggesting that glucosamine does not have a long-term impact on blood glucose.

Additionally, the consequences of glucosamine supply on the body weight of the mice were examined. As expected, a significant decrease in the body weight of the animals with diabetes compared to the non-diabetic controls was observed. Among the non-diabetic controls, glucosamine supplementation induced a significant 17% body weight gain (Figure 1C). However, no such change in weight was observed upon glucosamine supplementation in diabetic animals, suggesting a potential hyperglycemia-independent connotation.

In accordance with the known consequences of diabetes, water and food intake as well as urine and feces output of diabetic mice were significantly increased compared with non-diabetic controls. Despite the body weight gain in the non-diabetic animals, no changes in the food and water consumption or urine and feces output of the animals in the glucosamine-treated group were observed in either non-diabetic or diabetic animals (Supplementary Table 1). Additionally, we evaluated whether glucosamine supplementation causes liver damage by measuring the levels of activity of the enzymes glutamic oxaloacetic transaminase (GOT) and glutamic pyruvic transaminase (GPT) in the plasma. Glucosamine treatment did not alter GOT and GPT activity in non-diabetic or diabetic animals, indicating the safety of glucosamine at this dose for liver function (data not shown).

Finally, glucosamine levels in the plasma were determined by UPLC, showing a significant increase of glucosamine in the treated non-diabetic and diabetic mice. Interestingly, the plasma concentrations of glucosamine in the diabetic mice were much higher than those in the non-diabetic mice, while there was no difference in plasma glucosamine levels between diabetic and non-diabetic mice without glucosamine supplementation, suggesting a possible differential metabolism or distribution of exogenous glucosamine in diabetic individuals (Figure 1D).

### 3.2 Glucosamine exerts a neuroprotective effect in the diabetic retina

**Figure 2:**
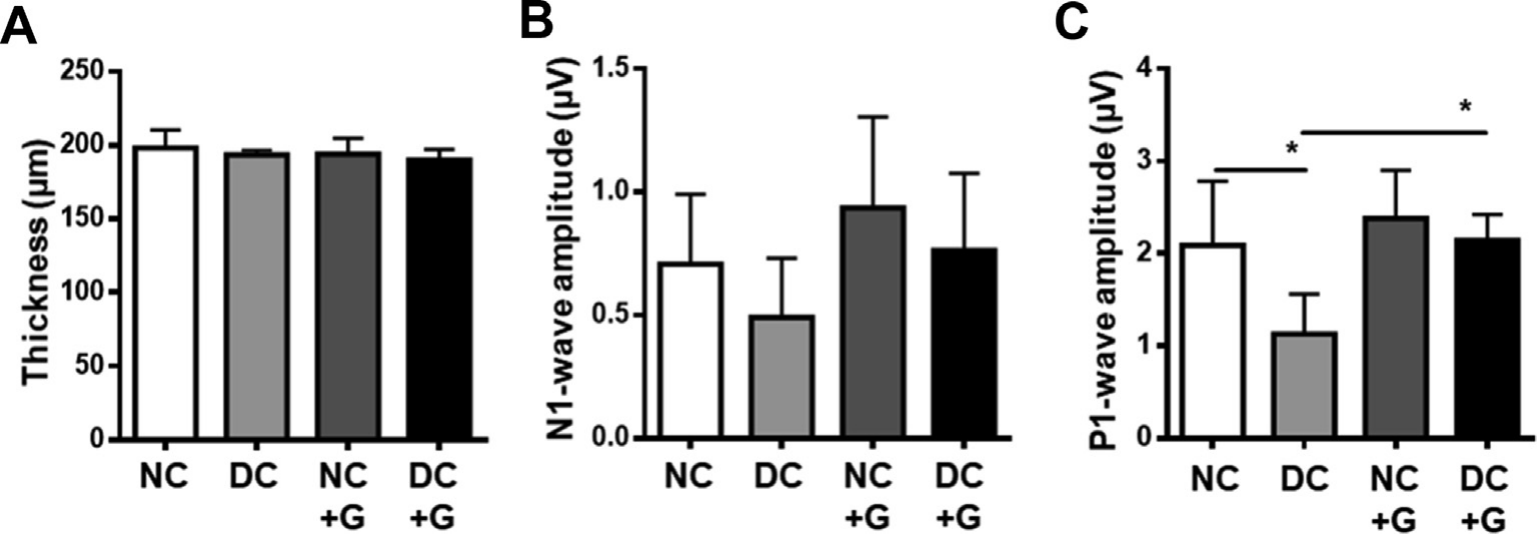
**Glucosamine is neuroprotective in the diabetic retina. A:** Optical coherence tomography of the retina was performed in non-diabetic and diabetic mice with and without 24 weeks of glucosamine supplementation to assess retinal layer thickness. **B:** N1-wave amplitude of the electroretinogram (ERG). **C:** P1-wave amplitude of the same ERG. n = 6–8, ∗p < 0.05.

Besides a damaged vasculature, early abnormal neuronal function is also a hallmark of diabetic retinopathy. In order to assess the impact of glucosamine on the neuronal aspect of the retina, morphological and functional tests were performed. Non-invasive optical coherence tomography (OCT) was used to measure the thickness of the mouse retinas. The retinal thickness remains unaltered between the groups, showing no changes with glucosamine supplementation (Figure 2A). Furthermore, we examined the thickness of individual retinal layers using paraffin sections, once again showing no significant changes between the groups (Fig. S1).

The electrical activity of the retina in response to a light stimulus, as assessed by electroretinography (ERG), gives an indication of the neuronal function of the retina. The different waveforms generated in the ERG correspond to the activity of different cell types in the retina. The N1-wave of the ERG, generated by photoreceptors, showed no significant changes between any of the groups under photopic conditions (Figure 2B). In the diabetic animals, the amplitude of the P1-wave is significantly reduced compared to the non-diabetic controls, indicating impaired function of inner retinal signal amplification, which under photopic conditions is mainly performed by ON bipolar cells. However, in diabetic animals an impairment of the P1-wave is more indicative of Müller cell dysfunction, as the Müller cells regulate the inner retinal signal transduction [[26], [27], [28]]. Glucosamine treatment did not affect the P1-wave in the non-diabetic animals. Under diabetic conditions, however, glucosamine was able to rescue the P1-wave amplitude, suggesting that it could play a role in the restoration of the neuronal function in the diabetic retina, possibly via its action on Müller cells (Figure 2C).

### 3.3 Glucosamine-induced neuroprotection is likely a result of diminished Müller cell activation

**Figure 3:**
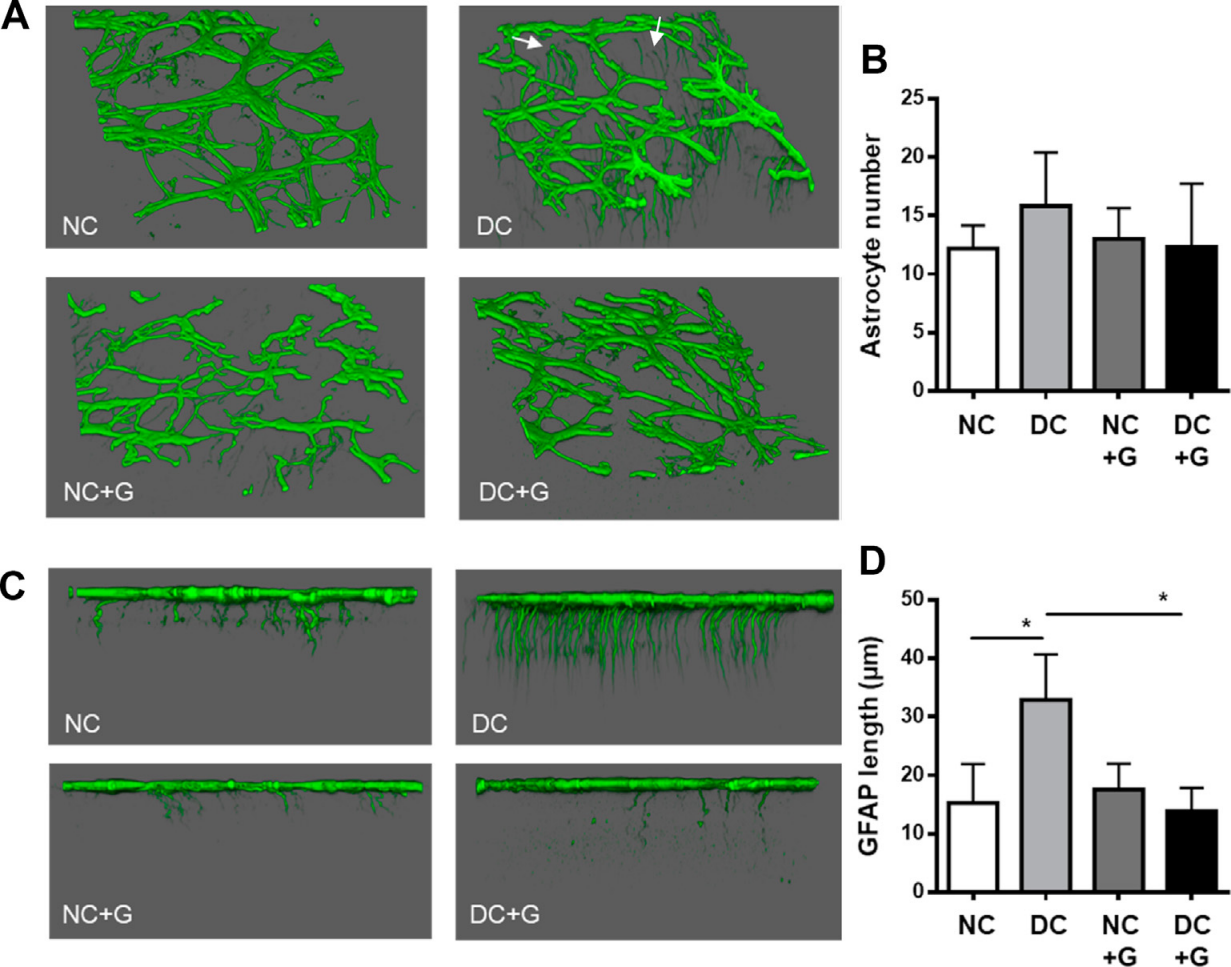
**Glucosamine reduces Müller cell activation in the diabetic retina.** Mice without and with diabetes induction were supplemented with glucosamine for 24 weeks and then sacrificed. Whole-mount retinas were stained for GFAP and analyzed by confocal microscopy. **A:** Topological 3D images showing astrocytes and the presence of Müller cell endfeet (indicated by arrowheads) in diabetic retinas. **B:** Quantification of astrocyte number in the superficial layer. **C:** Confocal z-stack topographical analysis displaying activated Müller cells rescued with glucosamine treatment. **D:** Quantification of the length of the GFAP, expressing Müller cells descending into the deep capillary layer in the retina (n = 3).

Müller cells are glial cells in the retina that perform several functions, including modulation of the neuronal microenvironment and facilitation of crosstalk between the vasculature and the neuronal compartment. We first examined the expression of GFAP in the retina. Under confocal microscopy, we reconstructed the three-dimensional architecture of GFAP-positive cells in the retina. In the superficial layer, astrocytes were stained positive for GFAP. In the non-diabetic retinas, GFAP expression can be seen mainly in astrocytes in the ganglion cell layer, which display normal morphology and are distributed evenly but sparsely throughout the retina (Figure 3A). In the diabetic retinas, however, the morphology of GFAP positive cells is slightly altered, displaying a more ramified structure. Through the astrocytes, the endfeet of the Müller cells are clearly visible, contrary to the non-diabetic retinas, confirming the activation of Müller cells as a hallmark of diabetic retinopathy (Figure 3A). In the diabetic and non-diabetic retinas treated with glucosamine, the astrocyte morphology resembles the normal phenotype seen in the non-diabetic retinas. There was no difference in the number of astrocytes between groups (Figure 3B). Following the GFAP expression in the endfeet down through the retina into the deep retinal layer, we saw increased GFAP expression throughout the Müller cells in the diabetic retinas, a phenotype of Müller cell activation. Glucosamine treatment rescued this activation of the Müller cells, seen by a significant reduction of GFAP staining beyond the superficial layer (Figure 3C,D), indicating that glucosamine treatment can reduce the activation of Müller cells in the diabetic retina.

**Figure 4:**
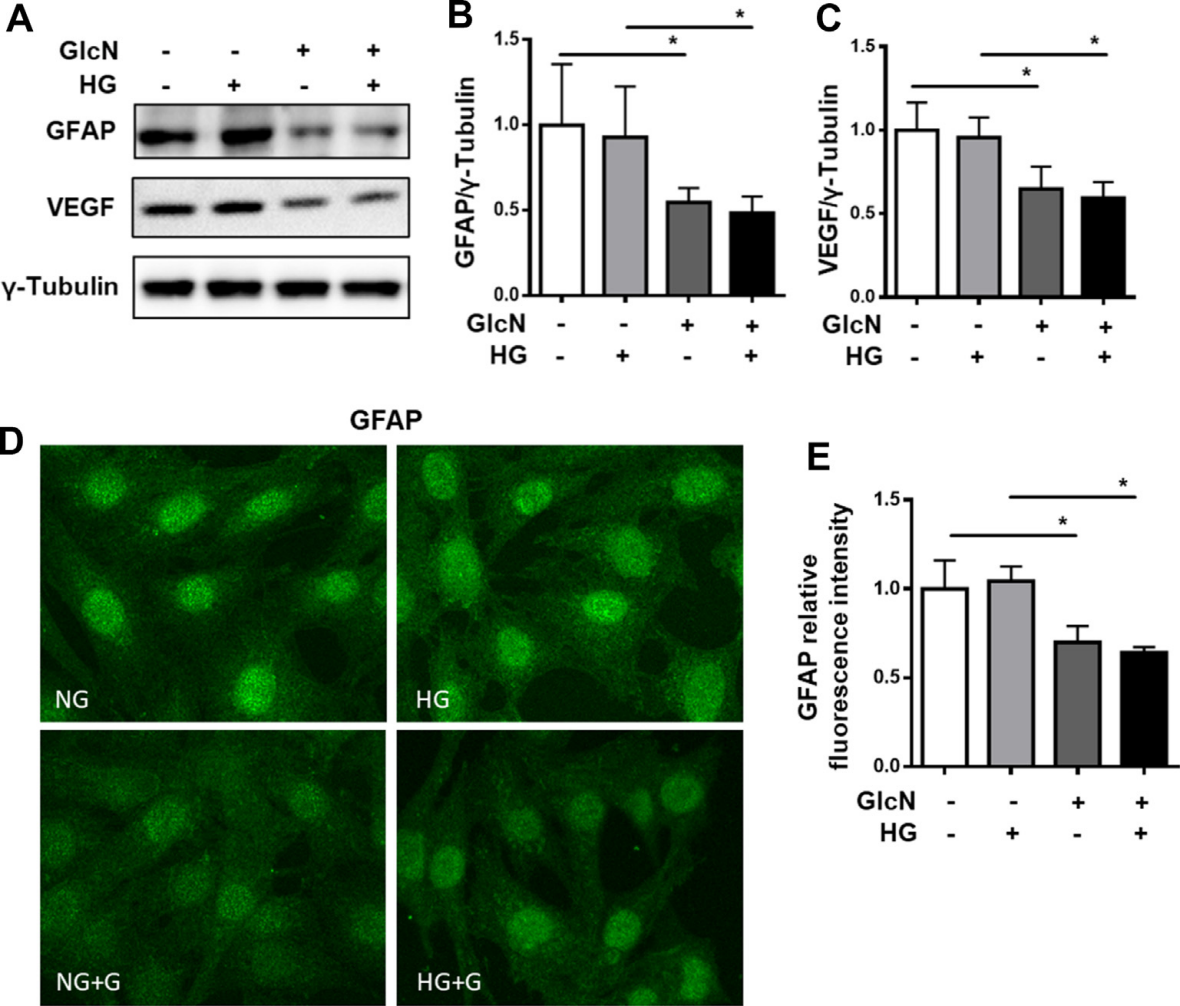
**Glucosamine reduces rat Müller cell activation.A:** Immunoblot analysis showing decrease in GFAP and VEGF levels with glucosamine treatment under normal and high-glucose conditions. Quantification of **B:** GFAP and **C:** VEGF with respect to γ-Tubulin; n = 6. **D:** Immunofluorescence staining of GFAP in rMCs and **E:** quantification of GFAP fluorescence intensity. n = 4, ∗p < 0.05.

Furthermore, using a rat Müller cell line (rMCs), we investigated the expression of GFAP with high glucose and glucosamine stimulation *in vitro* (Figure 4A,B). Strong GFAP expression was detected in the cultured rMCs in basal conditions. No significant effect of high glucose on the GFAP levels in the rMCs was observed. Glucosamine treatment resulted in a decrease in GFAP protein levels in both normal and high glucose–stimulated cells. Moreover, examining the expression of GFAP via immunofluorescence in the rMCs yielded similar results, showing that glucosamine can indeed reduce the GFAP expression in rMCs (Figure 4D,E).

In addition, we examined the expression of VEGF in the rMCs. Müller cells are a prominent secretor of VEGF in the retina, contributing to vascular regulation. HG did not alter VEGF expression compared to the normal glucose control. Glucosamine treatment was, however, able to significantly reduce the VEGF protein level in the rMCs (Figure 4A,C). The data hence indicate that glucosamine could exert a neuroprotective effect by inhibiting the activation of Müller cells, and may also contribute towards the dysregulation of vascular signaling via modulation of Müller cells.

### 3.4 Glucosamine prompts vascular damage in the retina

**Figure 5:**
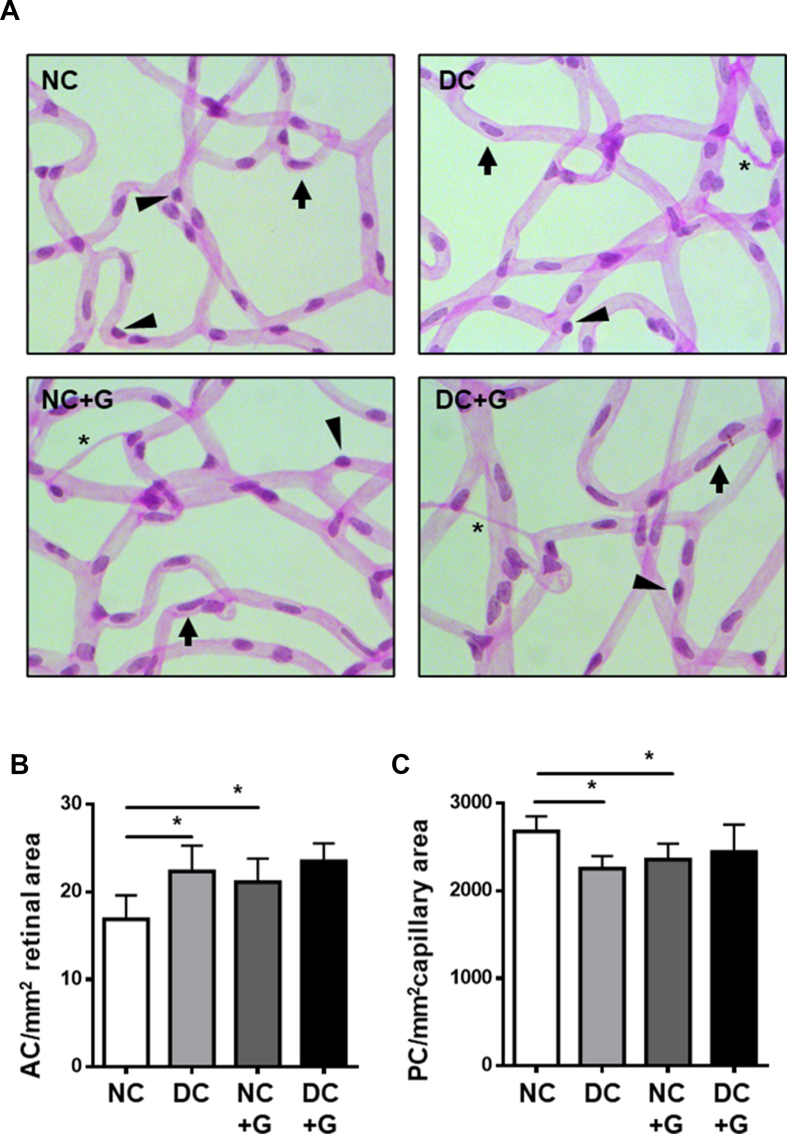
**Glucosamine exacerbates vascular damage in the retina. A:** Representative images of PAS staining of the retinal vasculature in the four groups shows stained vessels in the retina, with arrows indicating endothelial cells, arrowheads describing pericytes, and stars representing acellular capillaries. **B:** Quantification of acellular capillaries per mm^2^ retinal area. Diabetic retinas show increased AC formation; glucosamine treatment in non-diabetic animals also increases AC formation. **C:** Quantification of pericytes per mm^2^ capillary area. Diabetic retinas show increased PC loss; glucosamine treated retinas also display a decrease in PC coverage. n = 6–8, ∗p < 0.05.

Pericyte dropout and the formation of acellular capillaries are the hallmarks of vascular damage in the retina, and the quantification of their coverage in the retinal vasculature provides an indication of the initiation of retinal vascular injury. To assess the vascular damage in the retinas, the numbers of pericytes and acellular capillaries were quantified in retinal digest preparations stained with PAS (Figure 5A). Compared to the non-diabetic controls, the diabetic animals displayed a significant reduction in the number of pericytes (12%) and a significant increase in the formation of acellular capillaries (32%), confirming the occurrence of an early diabetic retinopathy phenotype of vascular damage. Surprisingly, under non-diabetic conditions, glucosamine supplementation induced a dropout of pericytes (16%) and an increase in acellular capillary formation (26%) similar to those observed in diabetic animals (Figure 5B,C). Compared to diabetic animals and to non-diabetic animals treated with glucosamine, supplementation in diabetic animals did not further increase the loss of pericytes or formation of acellular capillaries. Since glucosamine treatment induced vascular damage in non-diabetic animals but had no further damaging effects in diabetic animals, the data suggest that glucosamine may cause vascular damage in the retina.

### 3.5 Glucosamine induces suppression of VEGFR2 and Ang2 protein content in cultured endothelial cells

**Figure 6:**
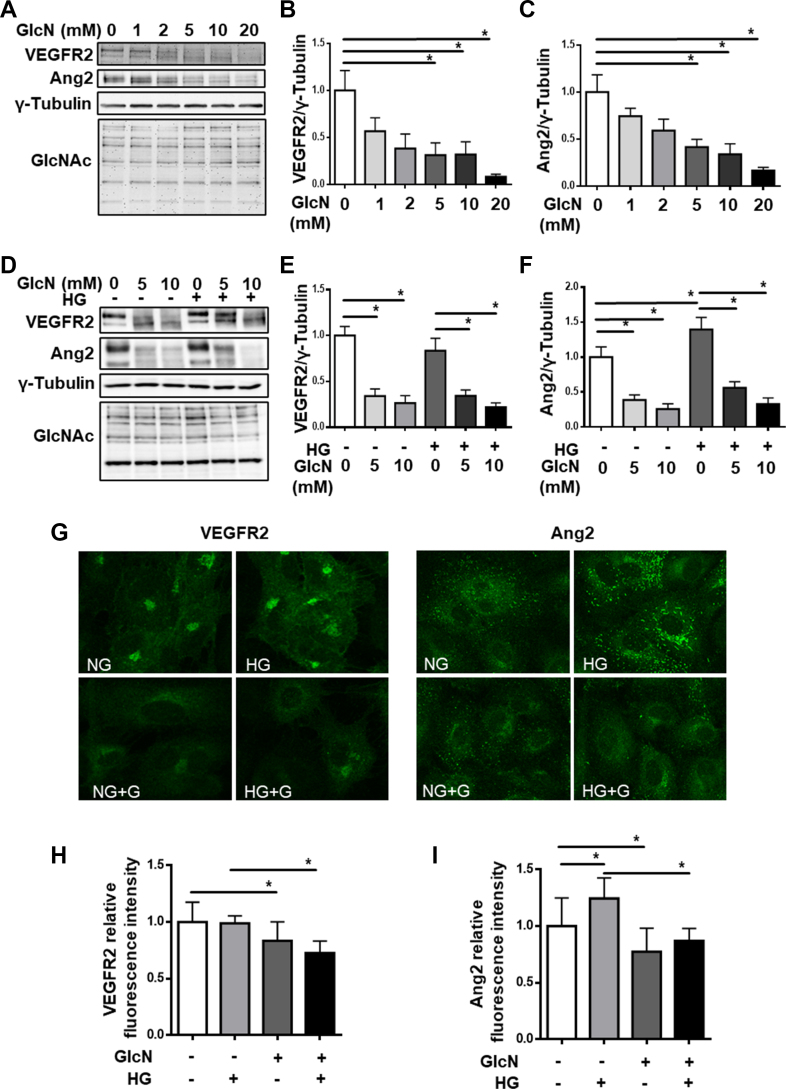
**Glucosamine dysregulates VEGFR2 and Ang2 protein expression in HUVECs. A:** Immunoblot analysis showing dose-dependent decreases in VEGFR2 and Ang2 levels with glucosamine treatment, but no changes in protein O-GlcNAcylation. Quantification of **B:** VEGFR2 and **C:** Ang2 with respect to γ-Tubulin. **D:** Immunoblot analysis showing decreases in VEGFR2 and Ang2 levels with glucosamine in both normal and high-glucose conditions. Quantification of **E:** VEGFR2 and **F:** Ang-2 with respect to γ-Tubulin. **G:** VEGFR2 and Ang2 immunofluorescence staining in HUVECs. Quantification of **H:** VEGFR2 and **I:** Ang2 fluorescence intensity, which show reduction with glucosamine treatment. n = 4–7, ∗p < 0.05.

In order to obtain insights into the vascular damage observed *in vivo* with glucosamine supplementation, we assessed the impact of glucosamine on cultured endothelial cells. VEGF-VEGFR2 signaling is crucial to endothelial cell survival. Additionally, Ang2, a key growth factor that mediates pericyte loss [29], is produced by endothelial cells. For our first endothelial cell model, we used cultured human umbilical vein endothelial cells (HUVECs). Upon treating the HUVECs with increasing concentrations of glucosamine, we observed a concentration-dependent decrease in VEGFR2 expression at the protein level. In addition, a slight shift in the apparent molecular mass of the band representing VEGFR2 in SDS-PAGE was observed in the groups treated with glucosamine. Glucosamine concentrations over 5 mM significantly suppressed VEGFR2 expression in HUVECs (Figure 6A,B). The cells were additionally treated with high glucose and glucosamine for 24 h. High glucose treatment did not influence VEGFR2 expression, but glucosamine treatment led to the reduction and band shift of VEGFR2 in both normal and high-glucose conditions (Figure 6D,E). In cultured HUVECs, however, we could detect neither an alteration in VEGF content and secretion (Fig. S2), nor alterations in the activation of AKT or ERK1/2 as judged by their phosphorylation status by glucosamine (Fig. S3). Correspondingly, there was no impairment of HUVEC viability by up to 10 mM glucosamine (Fig. S4).

Elevated production of Ang2 from endothelial cells is a cause for the pericyte loss in the retinal vasculature [29]. Thus, we further studied the effect of glucosamine on Ang2 regulation. Surprisingly, a strong inhibition of Ang2 expression was identified upon glucosamine treatment. Similar to VEGFR2, the inhibition is dose-dependent; glucosamine concentrations of 5 mM and above induced a significant decrease in Ang2 expression (Figure 6A,C). Nevertheless, a stronger band shift of Ang2 was seen compared to VEGFR2. As shown in our previous studies [22,30], high glucose resulted in an increase in Ang2 protein expression. However, the upregulated Ang2 was inhibited by glucosamine treatment in high glucose, similar to that in normal glucose (Figure 6D,F). Unexpectedly, we observed no significant difference in the level of protein O-GlcNAcylation in the endothelial cells with glucosamine treatment in normal versus hyperglycemic conditions.

We substantiated our findings with immunofluorescence staining of VEGFR2 and Ang2 in HUVECs under normal and high-glucose conditions (Figure 6G). As with the immunoblot, we observed no change in VEGFR2 immunofluorescence expression under high-glucose conditions. Glucosamine treatment, however, significantly diminished the VEGFR2 signal (Figure 6H). Ang2 immunofluorescence was significantly higher with high glucose, as expected, and a decrease in the signal was seen in both normal and high-glucose conditions with glucosamine treatment (Figure 6I), corroborating the immunoblot findings.

**Figure 7:**
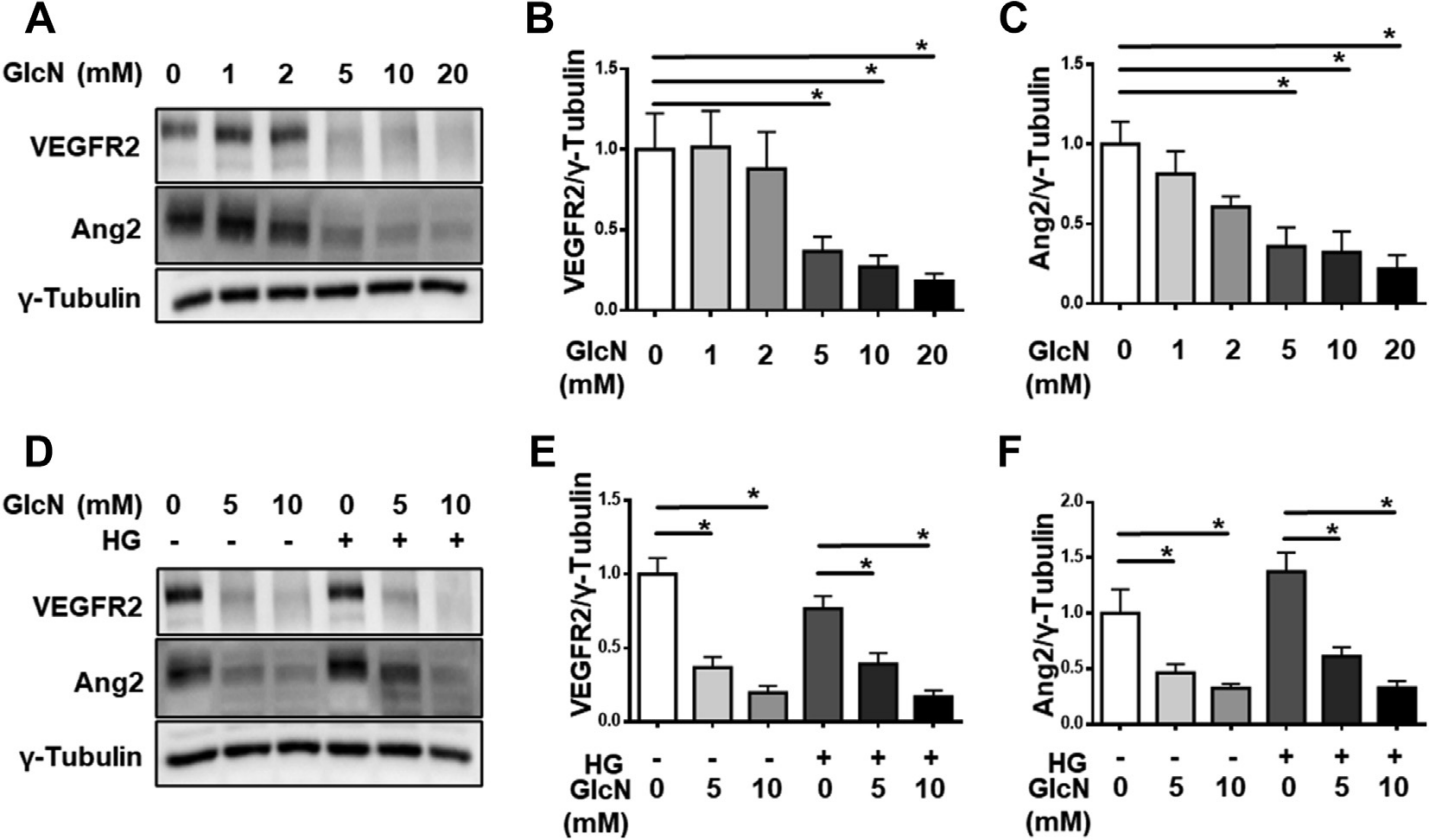
**Glucosamine dysregulates VEGFR2 and Ang2 protein expression in HRMVECs. A:** Immunoblot analysis showing dose-dependent decreases in VEGFR2 and Ang-2 levels with glucosamine treatment. Quantification of **B:** VEGFR2 and **C:** Ang-2 with respect to γ-Tubulin. **D:** Immunoblot analysis showing a decrease of VEGFR2 and Ang-2 levels with glucosamine in both normal and high-glucose conditions. Quantification of **E:** VEGFR2 and **F:** Ang-2 with respect to γ-Tubulin. n = 4–5, ∗p < 0.05.

In addition to HUVECs, we confirmed our findings in endothelial cells of retinal origin—human retinal microvascular endothelial cells (HRMVECs). The HRMVECs were treated with increasing doses of glucosamine, with or without high glucose. As in HUVECs, the HRMVECs showed a decrease in VEGFR2 (Figure 7A,B,D,E) and Ang2 (Figure 7A,C,D,F) protein levels as well as a corresponding band shift with increasing concentrations of glucosamine in both normal and high-glucose conditions. This indicates that the effect of glucosamine in endothelial cells is conserved across different endothelial beds, macro- and microvascular. In addition, since the HRMVECs are retinal in origin, the data suggest that a similar mechanism of action could occur in endothelial cells in the retina, perhaps contributing to the presence of the observed vascular damage with glucosamine.

## 4 Discussion

In our study, we demonstrate that glucosamine treatment in diabetic mice can improve the function of Müller glial cells, inducing a neuroprotective effect. However, it causes vascular damage in the retina, inducing pericyte loss and acellular capillary formation in non-diabetic retinas, which might be a result of interfering with endothelial survival signals. Our findings suggest that oral supplementation with glucosamine can elicit multiple countenances in the retina, including previously unknown risks.

To the extent of our knowledge, this is the first study demonstrating the effects of dietary glucosamine supplementation on neuronal and vascular function in diabetic retinopathy *in vivo*.

In accordance with previous studies [31,32], our experiments showed a decrease of the P1-wave amplitude in the retinas of diabetic animals, sustaining the neuronal damage characteristic in the diabetic retina. The protection from P1-wave impairment is most likely mediated by the protective effects on Müller cells. Dysfunction of glial cells promotes neurodegeneration; thus, proper function of glial cells plays a key role in neuroprotection [33,34]. Our findings support previously published reports. Hwang et al. have reported that glucosamine exerts a neuroprotective effect in a rat model of brain injury via suppression of the activation of microglial cells and inhibition of the release of inflammatory molecules [35]. Additionally, our results are consistent with a study conducted by Chen et al., who demonstrated that glucosamine treatment protects the retina against neuronal injury in an ischemia/reperfusion-induced rat glaucoma model by hampering damage to retinal ganglion cells [15]. However, our finding contrasts a study conducted by Nakamura et al., who showed that glucosamine supplementation results in the apoptosis of R28 cells, a retinal neuronal cell model used *in vitro* [36]. The discrepancy between the studies might be a result of different effects exerted by exogenous glucosamine on different cell types, since R28 cells are a retinal precursor cell possessing mainly neuronal cell properties with glial cell features.

Based on our data, which show an improved P1-wave amplitude following glucosamine treatment, we further focused on Müller cells in the retina. In accordance with published data [37], we found strong activation of Müller glial cells in the diabetic retina. Upon treatment with glucosamine, the GFAP expression in the Müller cells in the diabetic retinas was drastically reduced and bore closer resemblance to the non-diabetic retinas. Until now, there were no reports showing the glial response in the diabetic retina upon *in vivo* glucosamine treatment. Examining GFAP expression in a rat Müller cell line *in vitro*, we observed that glucosamine treatment reduces GFAP expression and hence Müller cell activation. Contrary to previous studies [38], we did not observe an increase in GFAP expression under high glucose conditions. This can likely be attributed to the fact that the Müller cell line *in vitro* was in a state of constant activation that could not be further stimulated by high glucose; glucosamine was nevertheless able to reduce this activation, resembling the reduced GFAP expression *in vivo*. Activation of Müller cells can have both beneficial and detrimental effects in the diabetic retina. Müller cells are the source of major survival signals for neurons in diabetes [39]. Activated glia can release neurotrophic factors and antioxidants, and hence contribute towards protecting the neurovascular unit. On the other hand, activated Müller cells can also produce proinflammatory cytokines that confer cytotoxic effects [40]. Our findings of reduced Müller cells activation combined with the ERG data indicating an improvement in the P1-wave amplitude suggest that glucosamine can ameliorate neuronal function in the diabetic retina by improving the function of Müller cells. In addition, glucosamine reduces the VEGF protein content in Müller cells, indicating a possible role in the modulation of survival signaling via Müller cells. In our study, however, we did not observe an alteration of protein O-GlcNAcylation by either high glucose or glucosamine treatment in Müller cells. Hence the neuroprotective effect of glucosamine is likely exerted via an HBP/O-GlcNAC–independent manner.

In opposition with the protective effects conferred by glucosamine on the neuronal function in the retina, its effects in the vasculature seem to be more detrimental. The damaging effect of glucosamine on the vasculature seen in our study supports studies showing that glucosamine consumption is correlated with the development of arteriosclerosis, CVD risk, and mortality, in which case endothelial dysfunction upon glucosamine supplementation may play a role [41]. In the non-diabetic animals, glucosamine induced the loss of pericytes and increased the formation of acellular capillaries. Pericyte loss in the diabetic retinas is triggered by increased levels of Ang2 [42]. Surprisingly, contradictory to these previous studies on diabetic retinopathy, glucosamine inhibits the protein expression of Ang2 *in vitro* in endothelial cells (both HUVECs and HRMVECs) despite the elevation of protein expression from high glucose, indicating that glucosamine-provoked vascular damage is presumably not initiated by pericyte loss. Ang2 regulation can be controlled by the cellular O-GlcNAc cycle [43]. However, no alteration of O-GlcNAcylation of proteins was identified in glucosamine-treated endothelial cells in our study. These results are inconsistent with other studies that show that glucosamine increases protein O-GlcNAc modification in endothelial cells [44], suggesting other possibilities of Ang2 regulation. Glucosamine, in addition to inhibiting Ang2 protein expression, also induces a band shift in Ang2, seen via immunoblot. This raises the possibility of Ang2 regulation via suppression of N-glycosylation in endothelial cells, similar to that in other cell types [45]. Ang2 can also perform as a survival factor in endothelial cells [46]. Vascular damage combined with lowered Ang2 expression could denote the impairment of survival signals caused by glucosamine in endothelial cells. Examining the main survival signals through VEGF-VEGFR2 in endothelial cells, we noticed that glucosamine starkly reduces the protein level of VEGFR2. It can therefore be speculated that glucosamine in the normal retina might interfere with VEGFR2-mediated survival signals in endothelial cells, and hence possibly contribute to the vascular damage. Nevertheless, in cultured endothelial cells, glucosamine did not alter VEGF content and secretion, the phosphorylation status of AKT and ERK1/2, or the cell viability under our experimental conditions, suggesting that the glucosamine effect on VEGFR2 occurs through a different mechanism. Because the cell viability with glucosamine concentration 25 mM and higher was significantly decreased, it is possible that under our experimental conditions, glucosamine interferes with VEGFR2 survival signals, but not enough to significantly affect cell viability.

Glucosamine treatment in our study did not induce changes in blood glucose or HbA1c. This information is in line with several clinical trials in humans that also show that glucosamine lacks an adverse impact on blood glucose and HbA1c levels [47] or on glucose metabolism [48]. The impact of glucosamine on body weight is controversial. A clinical study done on lean and obese subjects by Muniyappa et al. found no correlation between oral glucosamine supplementation and insulin resistance or endothelial dysfunction [49]. Ryczko et al. also reported no change in body weight upon glucosamine treatment in mice [50]. However, our findings confirmed a bodily weight gain following glucosamine treatment in non-diabetic mice, resonating with a study conducted by Hwang et al. reflecting similar weight gain in a rodent model, wherein they show that chronic activation of the HBP controls fat accumulation and insulin sensitivity [51]. A few studies have also reported side effects of glucosamine supplementation on the liver, leading to elevated levels of amidotransferases [52]; this phenomenon, however, was not seen in our study. Significantly elevated blood glucosamine levels detected in our study were still within the low range in mice. We assume free glucosamine does not occur in the HBP, but rather its derivatives, thus making it unlikely to cause toxic reactions. The results are in accordance with the report by Persiani et al., which shows that bioavailable glucosamine levels after supplementation are low and non-toxic [53]. It has been previously reported that endogenous glucosamine might be elevated in the serum of patients with diabetes [54]. The accumulation of glucosamine in the blood of diabetic mice treated with glucosamine is an indication of altered metabolism that might result from exogenous glucosamine supply in diabetic individuals. Glucosamine uptake into mammalian cells also occurs via glucose transporters [55,56]; hence, another possibility is that hyperglycemia could competitively inhibit the uptake of glucosamine into cells, thereby altering its distribution and increasing the plasma glucosamine level in diabetic animals treated with glucosamine.

We can therefore show that glucosamine exerts multifaceted effects on the retinal neurovascular unit, both protective and detrimental. It exerts a neuroprotective effect in the diabetic retina, but can induce vascular damage by suppressing the expression of VEGFR2 and Ang2 in normal retinas. As a widely used oral supplement, glucosamine requires caution for proper use. Further research needs to be performed in order to determine whether the benefits of glucosamine outweigh the risks.

## Author contributions

YF and TW conceived and designed the study. RE, LT, KM, MK, GJ, DZ, and GP performed experiments and acquired data. RE, YF, LT, MK, DZ, HPH, and MS analyzed and interpreted the data. RE and YF wrote the paper with input from the other authors. All authors gave final approval for publication.

## Funding

This work was supported by grants from the 10.13039/501100001659Deutsche Forschungsgemeinschaft (DFG GRK 1874-2 DIAMICOM, SP2 - Y.F., M.S., R.E. SP5 - H.P.H.).
