# Supplementary material for: Glucosamine protects against neuronal but not vascular damage in experimental diabetic retinopathy
Source: Mol Metab. 2021 Sep 20;54:101333. doi: 10.1016/j.molmet.2021.101333 (PMC8479835; doi:10.1016/j.molmet.2021.101333)
Supplement: Multimedia component 1 [file mmc1.docx]

|  | **NC** | **DC** | **NC+G** | **DC+G** |
| --- | --- | --- | --- | --- |
| **Water (ml)** | 2.28±1.12 | 16.36±5.71 | 2.00±0.61 | 17.22±6.30 |
| **Food (g)** | 0.91±0.73 | 4.16±0.64 | 0.77±0.24 | 4.00±0.87 |
| **Urine (ml)** | 0.57±0.41 | 13.50±4.45 | 0.72±0.38 | 14.18±5.55 |
| **Feces (g)** | 0.93±0.59 | 2.81±0.72 | 0.67±0.31 | 2.61±0.73 |

**Table S1: Glucosamine does not affect the water and food intake or urine and feces output of normal and diabetic mice**. Water and food intake and urine and feces output as measured over a 16 h period using a metabolic cage show increase in parameters under diabetic conditions, but no influence of glucosamine.


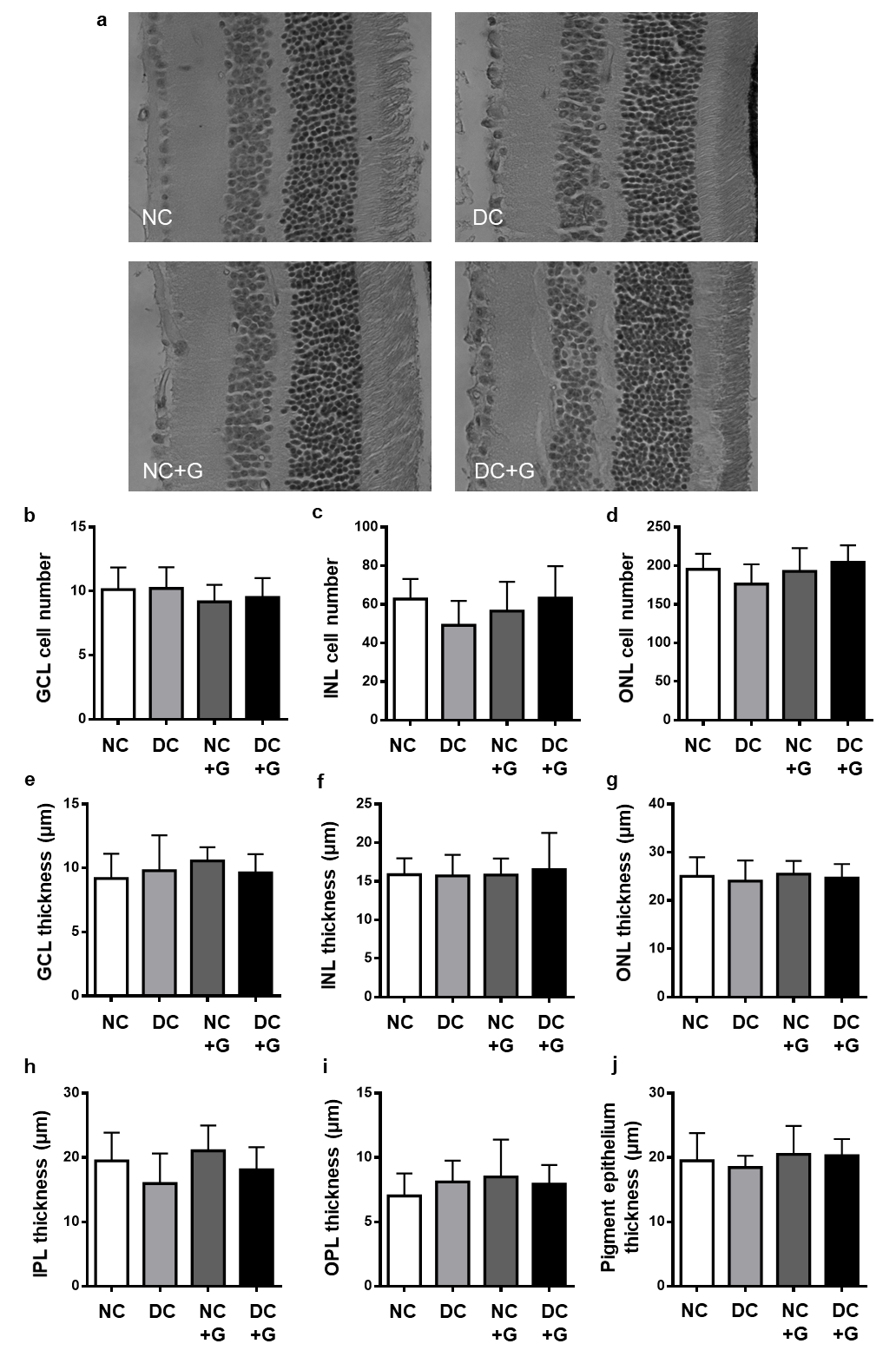


**Figure S1: Neuroretinal layer thickness is unaltered by glucosamine. a:** Representative images of retinal paraffin sections stained with Periodic Acid-Schiff displaying the layers in the neuroretina. Quantification of the cell number in **b:** ganglion cell layer (GCL), **c:** inner nuclear layer (INL), **d:** outer nuclear layer (ONL). Quantification of layer thickness of **e:** GCL, **f:** INL, **g:** ONL, **h:** inner plexiform layer (IPL), **i:** outer plexiform layer (OPL), and **j:** pigment epithelium showing no changes between the groups; n=3.


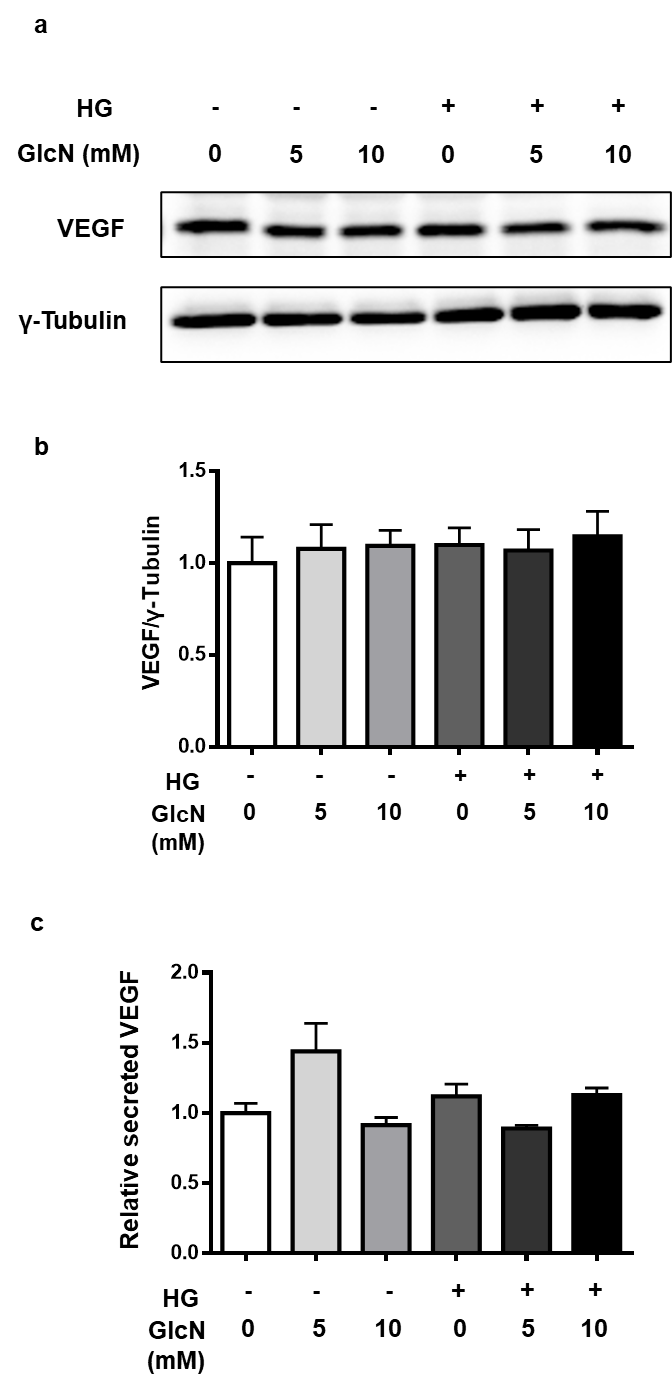


**Figure S2: Glucosamine does not influence VEGF in HUVECs. a:** Immunoblot analysis of VEGF with high glucose and glucosamine stimulation showing no changes with either treatment. **b:** Quantification of VEGF with respect to γ-Tubulin, n=3. **c:** Quantification of relative VEGF secretion in the supernatant of cultured HUVECs, measured via ELISA (R&D Systems), n=4.


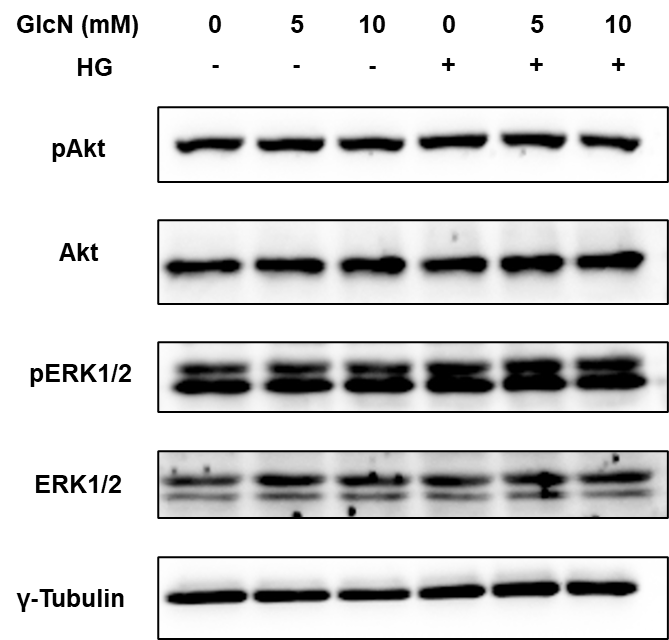


**Figure S3: AKT and ERK1/2 phosphorylation and total protein content is unaffected by glucosamine.** Immunoblot analysis of pAKT, AKT, pERK1/2, and ERK with γ-Tubulin as a housekeeping control protein showing no changes in either phosphorylation or total protein levels with high glucose and glucosamine stimulation.


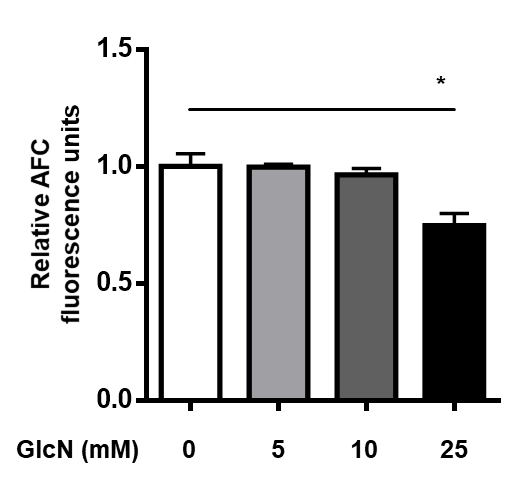


**Figure S4: Measurement of cell viability with glucosamine treatment.** HUVEC cell lysates were analyzed using the cell viability assay (Promega). Quantification of relative fluorescence units of AFC (aminofluorocoumarin) as an indicator of cell viability shows no change with 5 and 10 mM glucosamine but a decrease with 25 mM glucosamine, n=3.
